# Supplementary material for: Influence of Butorphanol, Buprenorphine and Levomethadone on Sedation Quality and Postoperative Analgesia in Horses Undergoing Cheek Tooth Extraction
Source: Vet Sci. 2022 Apr 6;9(4):174. doi: 10.3390/vetsci9040174 (PMC9029614; doi:10.3390/vetsci9040174)
Supplement: Supplementary file 1 [file vetsci-09-00174-s001.zip › vetsci-1641280-supplementary.pdf]

Supplemental material

Table S1. Sedation score (Assessment and adjustment of depth of sedation).

Score	Ataxia	Chewing on the mouth gag	Headshaking	Tongue activity	Defense behaviors towards manipulation
1	No ataxia	No chewing	No headshaking	No activity	No defense behaviour
2	Mild ataxia and swaying, occasionally laying against the stocks	Occasional chewing	Occasional headshaking	Occasional tongue activity	Mild defense behavior, no influence on extraction
3	Moderate ataxia, constant leaning against the stocks, buckling of limbs	Continuously chewing	Continuously mild headshaking	Continuous mild tongue activity	Moderate defense behavior, mild influence on extraction
4[a]	Severe ataxia, constant leaning against the stocks, permanent buckling of limbs [b]	Occasional severe chewing	Occasional severe headshaking	Occasional severe tongue activity	Severe defense behavior, moderate influence on extraction
5	Recumbency	Horse does not tolerate the mouth gag	Continuously severe headshaking, no manipulation possible	Continuously severe tongue activity, no manipulation possible	Manipulation not possible, horse does not tolerate instruments for extraction
Total score	1-5	1-5	1-5	1-5	1-5

[a] When one parameter was scored ≥ 4 an additional bolus of detomidine (3 µg/ kg IV) was administered and the detomidine infusion rate was increased by 10 µg/ kg/h. [b] When ataxia was scored ≥ 4, the infusion rate was decreased by 10 µg/ kg/h.
